# Factors Affecting Dental Caries Experience in 12-Year-Olds, Based on Data from Two Polish Provinces

**DOI:** 10.3390/nu14091948

**Published:** 2022-05-06

**Authors:** Kinga Andrysiak-Karmińska, Alicja Hoffmann-Przybylska, Piotr Przybylski, Zuzanna Witkowska, Ewa Walicka, Maria Borysewicz-Lewicka, Iwona Gregorczyk-Maga, Mansur Rahnama, Karolina Gerreth, Justyna Opydo-Szymaczek

**Affiliations:** 1 Department of Risk Group Dentistry, Chair of Pediatric Dentistry, Poznan University of Medical Sciences, 70 Bukowska Street, 60-812 Poznan, Poland; kandrysiak@ump.edu.pl (K.A.-K.); alicjahoffmann@ump.edu.pl (A.H.-P.); piotrprzybylski@ump.edu.pl (P.P.); ewa.nitschke@gmail.com (E.W.); klstomdz@ump.edu.pl (M.B.-L.); 2 Department of Pediatric Dentistry, Chair of Pediatric Dentistry, Poznan University of Medical Sciences, 70 Bukowska Street, 60-812 Poznan, Poland; zwitkowska@ump.edu.pl; 3 Department of Pediatric Dentistry, Jagiellonian University Medical College, 4 Montelupich Street, 31-155 Cracow, Poland; iwona.gregorczyk-maga@uj.edu.pl; 4 Faculty of Dentistry, Chair of Dental Surgery Department, Medical University in Lublin, 6 Chodzki Street, 20-950 Lublin, Poland; rahnama.m@interia.pl

**Keywords:** dental caries, dietary habits, adolescents

## Abstract

(1) Background: Dental caries is a chronic disease that affects a child’s dentition from the first stages of life. Several factors contribute to the development of the disease, including an improper diet. This cross-sectional study aimed to identify risk factors of dental caries in 12-year-old adolescents from Greater Poland and Lubusz Provinces (Poland). (2) Material and methods: The research was conducted in adolescents from five primary schools. A questionnaire consisted of close-ended questions on socioeconomic characteristics on family, diet, and oral hygiene habits. An assessment of the dentition was carried out in accordance with World Health Organization (WHO) recommendations. In addition to cavitated carious lesions, incipient caries lesions were noted according to the International Caries Detection and Assessment System, adapted for epidemiological studies (ICDASepiDMFt). (3) Results: The mean number of teeth with untreated caries; removed due to caries; and restored because of caries (DMFt) was 1.52 ± 1.90, while the ICDASepiDMFt index amounted to 2.64 ± 2.55, respectively. Children who did not brush every day had significantly higher odds of having ICDASepiDMFt > 0 than children brushing at least once daily (OR = 10.32, 95% CI = 1.36–78.32, p = 0.0240). Adolescents who drank sweet carbonated drinks every day had significantly higher ICDASepiDMTt than children who drank sweet carbonated drinks less frequently (p = 0.0477). (4) Conclusions: The research revealed that dental caries indices of 12-year-old adolescents from Greater Poland and Lubusz Provinces depend mainly on oral hygiene behaviors. The only significant nutritional factor that differentiated the caries intensity was the daily consumption of sweet carbonated drinks.

## 1. Introduction

Dental caries is a chronic disease that affects a child’s dentition from the first stages of life. When left untreated, the process can expand quickly and significantly damage primary and secondary dentition [1].

The disease develops due to a complex interaction between three essential factors: the microorganisms (the dental plaque), the substrate (the fermentable carbohydrates in a diet), and the host susceptibility (the teeth and saliva) [2]. Cariogenic microflora ferments dietary carbohydrates into acids that demineralize the dental hard tissues, i.e., enamel, dentine, and cementum [3]. Apart from the sugar intake, the frequency of meals, consistency and stickiness of food, duration of exposure, and type of carbohydrates influence dental caries risk [4]. At the same time, various factors may modify and counteract the effects of diet. They include oral hygiene practices, the use of fluoride and antimicrobial agents, and professional preventive treatments such as pits and fissures sealing [1,3,5,6,7]. Although the etiological factors of caries have been known for many years, the disease is almost omnipresent and affects patients of all ages [2]. It is noteworthy that the oral health status of the inhabitants of different European Union countries varies significantly. The declining prevalence of dental caries in most economically developed Western countries coincides with the disease’s high burden in several countries of Eastern Europe and Central Europe, including Poland [8,9].

Due to the complexity of the caries process, effective preventive programs must be adapted to the specific needs of the community they are to serve. A successful program in one population may not be very effective in another population with different cultural and behavioral habits and different access to fluoride and dental care [2,10].

Polish national oral health studies “Monitoring the oral health of the Polish population in 2016–2020” help the health policymakers assess country treatment needs and determinants of oral health periodically and introduce preventive actions [11]. Diet-related caries risk assessment is an essential part of the survey, and it is designed to identify improper and protective eating behaviors.

Although monitoring covers various age groups, children’s oral health should be treated as a priority research area. Children are the future of the generation, and many diseases, including dental caries, have their roots in childhood and adolescence [12,13]. Unhealthy eating behaviors and food choices such as preferences for sweetened food and soft drinks are common among these vulnerable groups, increasing the risk for caries development [14]. It must be remembered that dietary habits are formed in childhood. Children who consume free sugars frequently since the early years of childhood are more likely to follow a cariogenic diet rich in approachable, simple sugars and develop a sweet tooth in their adolescent and adult life [15].

In children with caries, the perception of sweet taste is characterized by lower sensitivity to sucrose than in children without caries [16]. Additionally, in children with caries, there is a strong positive correlation between the perception of sweet taste and the intensity of caries, and the frequency of eating sweets [16].

The age of 12 was determined as the age of global monitoring of dental caries for international comparisons and the evaluation of disease trends. In many countries, it is the last age at which data can be easily obtained through a reliable sample of the school system. Moreover, at this age, all the permanent teeth, except third molars, are usually erupted [17].

Thus, the study aimed to identify the dietary habits and their association with dental caries, in 12-year-old adolescents from Greater Poland and from Lubusz Provinces in Poland.

## 2. Materials and Methods

### 2.1. Ethical Issues

Before the study, the researchers obtained consent from the heads of schools and adolescents’ parents/legal caregivers and approval from the Warsaw Medical University Bioethical Committee (resolution no. KB/135/2019).

### 2.2. Study Participants

The study was performed among 12-year-old adolescents, i.e., six-grade students of five primary schools in two western provinces of Poland (Lubusz Province and Greater Poland Province) between September 2019 and November 2019. The research was a part of the Monitoring of Oral Health and its Determinants [11], a long-term project funded by the Polish Ministry of Health, and realized each year in different age groups of patients since 1997 [18,19].

Water in Poland is not artificially fluoridated. In most localities, fluoride concentration is below 0.5 mg/L [20]. According to data obtained in the sanitary station, the fluoride concentrations in the tap water drunk by study participants from Greater Poland and Lubusz Province were equal 0.26 and 0.36 mg/L, respectively.

The research included dental examination and a questionnaire study of adolescents. The selection of primary schools was carried out by a random sampling technique based on the school listings delivered by the Ministry of Education. The target schools were selected to ensure equal representation of inhabitants of urban and rural areas, i.e., two schools situated in an urban–rural commune in Lubusz Province, one school from the city, and two schools from villages in Greater Poland Province. In each school, out of the whole year, two classes of students were randomly chosen.

Considering the number of 12-year-old children in Lubusz and Great Poland Provinces in 2019 equaled 45,189 and 85% caries prevalence in this age group, at least 196 participants from both provinces constituted a representative sample size (assuming ±5% margin of error at a 95% confidence level).

The data concerning the project were presented to all parents/legal caregivers during the parent-teacher meetings. The examination was performed only in those adolescents who got written and informed consent for participation from their parents/legal caregivers. Full confidentiality of the gathered data was provided to all study participants.

Each student’s parent/legal caregiver, i.e., 110 persons in Lubusz Province and 274 in Greater Poland Province, was provided with a consent form.

Subsequently, students filled in questionnaires during classes at school and afterward delivered them to the teachers, who passed files on to the dentists. Altogether, 72 parents/legal caregivers from Lubusz Province and 152 from Greater Poland Province gave written and informed consent for their child’s questionnaire study and dental examination. However, 12 children were absent from school on the day of the examination, and 4 questionnaires were improperly filled out. Therefore, information concerning 16 students could not be analyzed due to numerous missing data. Hence, finally, 68 students in Lubusz Province and 140 adolescents in Greater Poland Province had dental examinations done and participated in the questionnaire study. Therefore, 208 sets of students’ dental charts and filled questionnaires were enrolled in the study.

### 2.3. Dental Examination

Teeth assessment was performed in accordance with recommendations introduced by the World Health Organization (WHO) [17]. Five dentists with similar clinical experience examined students at the school nurse’s office. Prior to the beginning of the study, all investigators performing a dental examination took part in a special training and calibration process prepared and carried out by pediatric dentists, i.e., the staff of Warsaw Medical University.

The training was done using pictures of various clinical situations and through the dental examination of fifteen patients on two separate days, with a one-week interval between such sessions. Two days prior to the examination of students at the schools, the training and calibration were repeated at the Chair of Pediatric Dentistry of Poznan University of Medical Sciences by two dentists who are experienced specialists in pediatric dentistry. The credibility of the clinical assessment of the teeth performed by investigators was verified based on re-examinations of twenty adolescents from the study sample [17]. Essential inter-examiner and intra-examiner agreements for diagnosing dental caries were obtained, with Cohen’s κappa values > 0.70.

Dental examination was performed using a standard dental probe (WHO probe) and plain dental mouth mirror under identical light conditions, i.e., using artificial light from a headlamp.

Before the evaluation, the teeth were not additionally cleaned and/or dried. Five surfaces were assessed and recorded for posterior teeth, i.e., mesial, distal, buccal/facial, lingual/palatal, and occlusal. Four surfaces were examined and recorded for anterior teeth, i.e., mesial, distal, facial, and palatal/lingual. Apart from the inter-proximal surfaces with no access, each surface of the entire dentition was rated. A probe was used only to verify the visual evidence of carious lesions. An individual permanent tooth was evaluated and scored as healthy, decayed (Dt), extracted because of caries (Mt), or filled due to caries (Ft). Based on the data obtained, the DMF index was calculated. This index expresses dental caries experience, and it is the sum of Dt, Mt, and Ft. The tooth was considered to have a carious cavity (Dt) when it demonstrated a detectably unmistakable cavity, softened area, or undermined enamel [17]. A tooth was considered carious when there was a carious cavity or secondary caries seen next to the filling (Dt). A tooth with a filling and carious lesion or temporary filling was also estimated as a carious tooth (Dt). The tooth was registered as missing (Mt) when it was extracted due to complications of the carious process (verified by an interview). When any doubt concerning reasons for extraction appeared, the missing tooth was not included in the index. The tooth was assumed as filled one (Ft) when at least one permanent restoration was placed for carious lesion treatment.

The coding system for each surface of the permanent tooth was as follows: 0 = no caries, 1 = carious cavity, 2 = secondary caries, 3 = filling, and 4 = extracted due to caries. Finally, based on a clinical dental examination, the number of teeth with carious cavities (Dt) and those extracted (Mt) and filled (Ft) due to the carious process were determined.

In addition to cavitated carious lesions, incipient caries lesions were noted (code 10 in the assessment chart) according to the International Caries Detection and Assessment System (ICDAS), adapted for epidemiological studies, which is compatible with the conventional WHO criteria. With the use of the simplified version adapted for the epidemiological studies with no availability of compressed air, the caries experience (including missing and filled surfaces/teeth) can be expressed conventionally (conventional DMFt), where the component “Dt” includes ICDAS moderate and extensive caries stages with the ICDASepiDMFt system, including ICDAS initial caries stages (those clinically visible without air-drying, namely, all ICDAS codes 2 white and brown, and ICDAS codes 1 brown) [21,22].

After the dental evaluation, each student was provided with instructions concerning oral hygienic and dietary habits. All parents/caregivers received the data concerning the oral health status of their children, and they were informed about the need for dental treatment in their children.

### 2.4. Socio-Medical Study of Students

The socio-medical study was performed using a questionnaire in the Polish language. The form had been previously utilized in Polish national oral health surveys as a part of the Monitoring of Oral Health and its Determinants [19]. The project follows the guidelines established by the WHO, which recommends the utilization of simplified structured questionnaires to gather information concerning oral health, with items adapted to national specificity [17,23].

Closed-ended questions concerned demographic and social characteristics such as the age of an adolescent; his/her sex; his/her parents’ education (primary; vocational; secondary with certificate of secondary school-leaving examination; or higher or incomplete higher, i.e., studies or college); his/her place of residence (city/countryside), including province (Lubusz/Greater Poland); his/her socioeconomic status (in the student’s opinion—very good/average/sufficient); information on whether the adolescent uses private dental service (yes/no); and data on the student’s daily consumption of raw vegetables and fruit (yes/no), daily consumption of sweets (including cookies, cakes, candies, and donuts) (yes/no), snacking habits regarding sugar-containing products at bedtime (yes/no), consumption of sweetened juices (several times per day/up to once a day), daily consumption of sweet carbonated beverages (yes/no), consumption of water or unsweetened tea (several times per day/less frequently), consumption of milk without sugar, consumption of natural yogurts (several times per day/maximum once a day), daily consumption of unsweetened chewing gums (yes/no), number of meals per day (up to 3/>3), snacking habits between the five main meals (yes/no), frequency of toothbrushing (at least twice per day/less frequently and at least once per day/less frequently), use of dental floss (yes/no), use of fluoridated toothpaste (yes or I am not sure/no), and participation in a school-based program of toothbrushing with fluoride gel (yes/no or I do not remember).

Finally, 208 sets of data concerning the clinical examination of the students’ teeth and questionnaires were gathered. Sixty-eight (32.69%) of 12-year-old students (41 females and 27 males; 37 adolescents from the city; and 31 individuals residing in the village area) were examined in Lubusz Province. In Greater Poland Province, 140 (67.31%) schoolchildren (71 females and 69 males; 71 from the city; and 69 from the countryside) were included in the study.

The inclusion and exclusion criteria for 12-year-old adolescents in the study are presented in Table 1, whereas in Table 2, the characteristics of the study group are shown.

After teeth evaluation, the researchers conducted oral health education sessions for all participants, providing oral health and toothbrushing training information. Additionally, brochures with data on the principles of proper oral hygiene and diet, the obligation to attend the dentist systematically, and the possibility to have a dental visit covered by the National Health Fund were spread among children’s parents/legal caregivers.

### 2.5. Statistical Analysis

The obtained data were statistically analyzed using MedCalc^®^ Statistical Software version 20.013 (MedCalc Software Ltd., Ostend, Belgium; https://www.medcalc.org; 2021, accessed on 10 April 2022), assuming a statistical significance level of *p* < 0.05.

The Shapiro–Wilk test was used to check the data distribution. The unpaired *t*-test and Mann–Whitney U test was used to compare quantitative variables (DMFt numbers and ICDASepiDMFt numbers), and the chi-square test was used to compare categorical variables. Factors contributing to the development of dental caries (DMFt > 0 and ICDASepiDMFt > 0) and relationships between them were assessed using odds ratios (OR) with 95% confidence intervals (CI).

## 3. Results

Table 3 presents the results of the questionnaire study. The DMFt values and ICDASepiDMFt (including initial-epiD) are depicted in Table 4. The mean numbers of teeth with untreated moderate/extensive caries (Dt), removed due to caries (Mt), and restored because of caries (Ft) were 0.79 ± 1.35, 0.02 ± 0.14, and 0.73 ± 1.26, respectively. The mean number of teeth with untreated caries (including initial-epiD (ICDASepiDt) was 2.64 ± 2.55. The mean numbers of DMFt and ICDASepiDMFt were 1.52 ± 1.90 and 2.64 ± 2.55, respectively. Caries frequency, defined as the percentage of subjects with DMFt > 0, was 55.8%. When incipient caries lesions detectable without drying were included, the caries frequency was 72.1%.

There were no significant predictors of DMFt in the statistical analysis, although the differences between the DMFt numbers of children coming from the families with different levels of education and between DMFt numbers of females and males were close to significant (*p* = 0.0786 and *p* = 0.0714, respectively). An analysis of ICDASepiDMFt numbers revealed that children brushing their teeth a maximum of once a day had significantly higher odds of having ICDASepiDMFt > 0 than children brushing their teeth twice daily (OR = 1.94, 95% CI 1.02–3.70, *p* = 0.0424), while children who did not brush their teeth every day had significantly higher odds of having ICDASepiDMFt > 0 than children brushing at least once daily (OR = 10.32, 95% CI = 1.36–78.32, *p* = 0.0240). Drinking water or unsweetened tea was associated with lower (close to significant) odds of having ICDASepiDMFt > 0 (OR = 0.58. 95% CI= 0.31–1.07, *p* = 0.0803). Children who brushed their teeth at least twice a day had significantly lower ICDASepiDMTt than children who brushed less frequently (*p* = 0.0003). Children who drank sweet carbonated drinks every day had significantly higher ICDASepiDMTt than children who drank sweet carbonated drinks less frequently (*p* = 0.0477) (Table 5).

The daily consumption of raw vegetables and fruit, drinking water, or unsweetened tea several times a day was associated with higher odds of twice daily toothbrushing (OR = 1.99, 95% CI = 1.13–3.50 and OR = 1.82, 95% CI = 1.04–3.18, respectively). Snacking sugar-containing products at bedtime was related to significantly lower odds of twice daily toothbrushing (OR = 0.24, 95% CI = 0.13–0.47), while this relationship was close to significant for daily consumption of sweet carbonated beverages (OR = 0.57, 95% CI = 0.29–1.11). Children who reported the use of dental floss had significantly higher odds of twice daily toothbrushing (OR = 2.21, 95% CI = 1.23–3.96) (Table 6).

## 4. Discussion

For many years, the consumption of nutritional sugars was considered one of the most critical risk factors for dental caries development. However, as stated at the FDI’s Second World Conference on Oral Health Promotion, where adequate oral hygiene and fluoride are present daily, diet has become a lesser factor in caries prevention [24,25].

At the same time, research on the influence of diet on caries development is continuing, and many reports confirm the relationship between oral health and nutrition [3,4,14,26,27].

Our study indicates that the percentage of 12-year-old Polish adolescents from Lubusz and Greater Poland Provinces with caries experience was 55.8%, and the mean DMFt amounted to 1.52. For comparison, in the other European countries, 12-year-old adolescents had lower DMFt numbers. DMFt indices ranged from 0.4 in Denmark in 2014, through 0.5 in Germany in 2014, 0.6 in the Netherlands in 2012, 0.7 in the United Kingdom in 2011 and Spain in 2014, to 0.8 in Sweden in 2011 [28]. Romanian adolescents had a higher DMFt index, which amounted to 3.13 (2020), and higher caries prevalence (95.5%) [28] compared to adolescents in our research. Similarly, Albanian and Croatian 12-year-old adolescents had higher DMFt indices (3.7 and 4.8, respectively) [29] than Polish children. This indicates that there are still significant differences between European countries. The average DMFt scores recorded in 12-year-old children in recent years during Polish monitoring studies (2.81 in 2014 and 3.75 in 2016) were higher than those observed in the present study [30]. This could be due to our study population coming from Western Poland, where the level of socioeconomic status is relatively high compared to other regions of the country [11].

In a study conducted in China on the population of students aged 12–14 years, the prevalence of dental caries and the DMFt index were comparable to those observed in our study and amounted to 56.9% and 1.45, respectively. As in our study, girls had a dental caries index higher than boys [31]. Other authors have reported similar results [32,33,34]. This might be due to the earlier eruption of teeth in females and prolonged exposure to the oral environment [33].

Since salivary secretion at night is low, the consumption of food and drinks at bedtime disturbs the balance between demineralization and remineralization [35,36,37]. In a study conducted in the United Kingdom on a group of adolescents aged 11–12 years, the consumption of free sugars before bedtime was a significant risk factor for caries experience. Children who ate snacks before bedtime had DMFt indices more than twice as high as those who did not [35]. Similarly, the study involving younger children with primary dentition revealed that the frequency of consumption of snacks/drinks before bedtime is significantly associated with dmft scores (r = 0.547, *p* = 0.001) [37]. Our study did not confirm the association between snacking at bedtime and dental caries experience.

The protective effects of water against dental caries are related to the lack of sugar in this drink and were confirmed by some studies [10,38]. Sanders and Slade showed that dental caries prevalence was higher in adolescents who did not drink tap water frequently than in children who drank tap water regularly [38]. Statistically significant differences were found between the DMFt indices of children who declared bottled water as a primary source of drink and children who used other beverages as a primary source of fluid [10]. In our study, children who drank water or unsweetened tea several times per day had close-to-significantly-lower odds of having ICDASepiDMFt > 0 (OR = 0.58, CI 95% = 0.31–1.07).

Although the frequent consumption of food leads to a repetitive attack of carbohydrates and acids on dental hard tissues [27], the present study did not demonstrate the association between dental caries and the daily consumption of sweets. Furthermore, we did not find any adverse effects of frequent food consumption, although we analyzed the impact of two extreme variations in the number of meals: the dental health of children who eat snacks between the five main meals, as well as children who eat up to three meals a day.

At the same time, adolescents drinking sweet carbonated drinks every day had significantly higher ICDASepiDMTt than those who consume such products less frequently (*p* = 0.0477).

The study by Marshall et al. revealed that the increasing number of eating events and higher exposures to 100% juice as a snack significantly increased dental caries risk in children [39]. The research of Hu et al. revealed that 12-years-old students who consumed sugar-containing snacks and drinks at least once a day presented higher scores on the DMFt index and, in general, had a higher risk of caries [1]. According to the results of the study by Asawa et al., dmft and DMFt scores were relatively higher for 12- and 15-year-olds who consumed sugary substances (soft drinks, sweets/chocolates, or cakes/pastries) more than once/day than those who had less than once/day [40].

It is generally observed that sugar-sweetened beverages, i.e., mineral waters (sweetened but noncarbonated), soft drinks (soda or pop), sports (electrolyte) drinks, and cordials (sweet concentrates to which water is added), are frequently consumed in numerous countries, and their intake patterns have shown an increase over time [41]. Many studies have confirmed the harmful effects of these products on oral health [41,42,43,44]. Armfield et al. performed a study on Australian children and adolescents aged 5 to 16 years old and concluded that the increased intake of sugar-sweetened beverages was associated with individuals having more dental caries in both milk and permanent dentition. Moreover, they also noted that increased exposure to fluoridated public water reduced the hazardous effect of such beverage consumption on the occurrence of dental caries [41]. Mello et al. observed that the consumption of soft drinks derived from cola, sugared beverages, and food two or more times per week were significantly associated with having dental caries (DMFt > 0) or even having four or more teeth affected by this disease (DMFt ≥ 4) [44], in thirteen-year-old Portuguese schoolchildren. It should be added that, as in Poland, in Portugal, drinking water is not fluoridated [44]. The authors also identified additional risk factors for dental caries such as female sex, attending a public school, and having parents with low educational attainment.

The role of parents’ education in shaping the pro-health behavior of their children has been confirmed in many studies [11,44,45,46]. Children’s oral health depends on the awareness of caregivers who are responsible for monitoring the child’s eating habits, oral hygiene, and regular visits at the dental office [11,29,47]. In our study, children of parents with university education had lower DMFt numbers than children from less-educated families (*p* = 0.0786, close to a significant difference).

The analysis of the ICDASepiDMFt results of our research revealed that children who brushed their teeth a maximum of once daily had significantly higher odds of having ICDASepiDMFt > 0 than those carrying out this procedure twice a day (OR = 1.94, 95% CI 1.02–3.70, *p* = 0.0424), whereas schoolchildren who did not brush their teeth every day had significantly higher odds of having ICDASepiDMFt > 0 than those brushing their teeth at least once daily (OR = 10.32, 95% CI = 1.36–78.32, *p* = 0.0240). Moreover, the individuals who brushed their teeth at least twice a day had significantly lower ICDASepiDMTt than children brushing their teeth less frequently **(***p* = 0.0003). This is in accordance with the study of Gupta et al. performed on 12-year-olds from Mathura City in India [48]. The authors showed, using multiple linear regression analysis for the determination of the independent effects of daily sugar intake, body mass index (BMI), and oral hygiene status on dental caries prevalence, that the status of oral hygiene had a significant effect on caries prevalence (OR = 5.061, *p* = 0.004, S). Additionally, they found that daily sugar intake and BMI had no significant effect on caries prevalence.

Good oral hygiene seems to be an essential factor for oral health [48]. Improper hygiene causes dental plaque accumulation, which is an important risk factor in the etiology of dental caries. Furthermore, the maintenance of good oral hygiene and the frequent application of fluoride allows one to keep one’s teeth intact, even when carbohydrate-containing food is often consumed [48]. In addition, Burt and Pai [49], in their systematic review concerning sugar consumption and caries risk, reported that the relationship between carbohydrate intake and dental caries is much weaker in the modern age of fluoride exposure than it was previously. At present, fluoride is widespread in toothpaste and professional applications, drinking water, and processed drinks and foods [49]. Although children in our study did not consume fluoridated water, most were exposed to fluoridated toothpaste and took part in the national school-based toothbrushing program with fluoride gel. Unfortunately, the exact data concerning the utilization of fluoride toothpaste in the examined population are unknown because many children (54.8%) were not aware of the fluoride content in the dentifrice. In the statistical analysis, we assumed that children who did not know whether their toothpaste contained fluoride belonged to the group using regular fluoridated toothpaste. In contrast, children who were sure to use fluoride-free toothpaste probably came from families of fluoride opponents. A previous study concerning children from the Greater Poland Province indicates that 3% of the families do not use fluoride products [10]. In recent years, we have observed a growing problem of fear of fluoride compounds, analogous to the anti-vaccination movement that pediatrics struggle with.

It is noteworthy that health-related habits might be related to each other, and the oral health status can be a result of an interplay between them. Asawa et al. noticed that children who did not consume fresh fruits and vegetables daily showed greater consumption of soft drinks. Oral health habits such as tooth brushing frequency were significantly associated with chocolates and cake/pastries consumption (students brushing their teeth less than once a day consumed more sugar-containing products) [40]. Similarly, in our study, most of the dietary factors did not reach the threshold of a significant association with dental caries indices. However, some of them were significantly related to oral hygiene habits. Daily consumption of raw vegetables and fruit and drinking water or unsweetened tea several times a day were associated with significantly higher odds of twice daily toothbrushing, while snacking on sugar-containing products at bedtime and daily consumption of sweet carbonated beverages were related to significantly lower and close-to-significantly-lower odds of twice daily brushing.

The assessment of the etiological factors of dental caries is an important stage in the preparation of effective prophylactic programs, which should focus on potentially modifiable behavioral habits [50]. Since regular toothbrushing was proved to be the most effective method of dental caries prevention in the examined population, oral health programs at schools and health-promoting actions in both Polish provinces should focus on education and the motivation for oral hygiene procedures. Adolescents and their parents should be made aware of the role of oral hygiene in preventing tooth decay, and the harmful effects of having widely available sweet carbonated drinks. They should also be encouraged to visit the dental office for regular dental check-ups [51].

It must be emphasized that problems caused by dental caries and its complications might be severe not only for oral health but also for the whole organism and general health, and for the quality of life of the children and their families [47,52]. Issues caused by the disease involve pain, psychological and speech difficulties, and problems with food consumption and are a common reason for absence from school [52].

Our study is not free of limitations. Firstly, the data related to risk factors were obtained through a questionnaire, and children might not have provided complete information. The clinical examination was carried out without any additional diagnostic aids, e.g., x-rays, which could affect the accuracy of dental health assessment. The dental examination took place in schools without the presence of parents/legal guardians. Therefore, it was not possible to refer children for additional tests.

On the other hand, some strengths of this research should be underlined. All five examiners involved in the study were trained and calibrated. Thus, the accuracy of the research was assured.

In summary, dental caries lesions and restorations were found in significant numbers of teeth in examined adolescents. Therefore, it is imperative to enable the accessibility of the parents/legal caregivers and staff of the schools to dental-health promotion programs, with a particular stress on oral hygiene habits.

## 5. Conclusions

The research revealed that dental caries indices of 12-year-old adolescents from Greater Poland and Lubusz Provinces depend mainly on oral hygiene behaviors. The only significant nutritional factor that differentiated the caries intensity was the daily consumption of sweet carbonated drinks.

## Figures and Tables

**Table 1:** The inclusion and exclusion criteria for 12-year-old students participating in the study.

Inclusion Criteria	Exclusion Criteria
Age between 12 years and 12 years 11 months	Different ethnic or national background
Polish nationality	Child’s uncooperativeness during dental examination
Place of residence: Lubusz and Greater Poland Provinces	Questionnaires improperly filled in by students and returned to examiners
Parental written and informed consent for dental examination and questionnaire study of the child	
Students present at school on days of dental examination and questionnaire study	

**Table 2:** The demographics of the study group.

Subjects’ Characteristics		*N* (%)
		208 (100.0)
Parents’ education	neither parent has university/college education	127 (61.06)
	at least one parent with university/college education	81 (38.94)
Sex	females	112 (53.85)
	males	96 (46.15)
Place of residence	city	108 (51.92)
	countryside	100 (48.08)
Province	Lubusz	68 (32.69)
	Greater Poland	140 (67.31)
Socioeconomic status (in the student’s opinion)	very good	94 (45.19)
	average or sufficient	114 (54.81)

**Table 3:** The results of the questionnaire study.

Subjects’ Characteristics		*N* (%)
		208 (100.0)
Visitors of private dental office	yes	120 (57.69)
	no	88 (42.31)
Daily consumption of raw vegetables and fruit	yes	126 (60.58)
	no	82 (39.42)
Daily consumption of sweets (including cookies, cakes, candies, and donuts)	yes	73 (35.10)
	no	135 (64.90)
Snacking on sugar-containing products at bedtime	yes	138 (66.35)
	no	70 (33.65)
Drinking of sweetened juices	several times/day	20 (9.61)
	up to once a day	188 (90.39)
Daily consumption of sweet carbonated beverages	no	165 (79.33)
	yes	43 (20.67)
Drinking water or unsweetened tea	several times/day	98 (47.11)
	less frequently	110 (52.89)
Milk without sugar, natural yogurts	several times/day	31 (14.90)
	maximum once a day	176 (84.62)
Daily consumption of unsweetened chewing gums	yes	38 (18.27)
	no	170 (81.73)
Number of meals/daily	up to 3	31 (14.90)
	>3	177 (85.10)
Snacking between 5 main meals	yes	123 (59.13)
	no	85 (40.87)
Frequency of toothbrushing	at least twice/day	120 (57.69)
	once daily	64 (30.77)
	less frequently	24 (11.54)
The use of dental floss	yes	81 (38.94)
	no	127 (61.06)
The use of fluoridated toothpaste	yes	83 (39.90)
	I am not sure	114 (54.81)
	no	11 (5.29)
Participation in school-based program of supervised toothbrushing with fluoride gel	yes	127 (61.06)
	no or I do not remember	81 (38.94)

**Table 4:** Caries frequency, DMFt index (moderate/extensive D), ICDASepiDMFt (including initial-epiD), and its components in examined population.

	Dt	Mt	Ft	DMFt	DMFt > 0 n (%)	DMFt = 0 n (%)
mean ± SD	0.79 ± 1.35	0.02 ± 0.14	0.73 ± 1.26	1.52 ± 1.90	116 (55.8%)	92 (44.2%)
range	0–8.00	0–1.00	0–6.00	0–8.00		
	ICDASepiDt	Mt	Ft	ICDASepiDMFt	ICDASepiDMFt > 0 n (%)	ICDASepiDMFt > 0 n (%)
mean ± SD	1.97 ± 2.32	0.02 ± 0.14	0.73 ± 1.26	2.64 ± 2.55	150 (72.1%)	58 (27.9%)
range	0–10.00	0–1.00	0–6.00	0–10.00		

**Table 5:** An analysis of the factors associated with the development of dental caries in the examined children.

Factor	Categories of Response	DMFt	DMFt > 0	DMFt = 0	The Odds Ratio of DMFt > 0		ICDASepiDMFt > 0	ICDASepiDMFt = 0	The Odds Ratio of ICDASepiDMFt > 0
						ICDASepiDMTt			
		Mean ± SD	n (%)	n (%)	OR (95% CI)	Mean ± SD	n (%)	n (%)	OR (95% CI)
Parents’ education	neither parent has university/college education	1.71 ± 2.04	74	53	1.30 (0.74–2.27)				
						2.86 ± 2.32	94	33	1.07 (0.80–1.28)
	at least one parent with university/college education	1.24 ± 1.62	42	39	1				
						2.30 ± 2.68	56	25	1
		*p* = 0.0786 *			*p* > 0.05	*p* > 0.05			*p* > 0.05
Sex	females	1.73 ± 1.97	68	44	1.55 (0.89–2.68)	2.52 ± 2.46	81	31	1.02 (0.56–1.88)
	males	1.28 ± 1.78	48	48	1	2.78 ± 2.67	69	27	1
		*p* = 0.0714 *			*p* > 0.05	*p* > 0.05			*p* > 0.05
Place of residence	city	1.41 ± 1.86	58	50	0.84 (0.49–1.45)	2.30 ± 2.45	82	26	1.48 (0.81–2.73)
	countryside	1.65 ± 1.95	58	42	1	2.95 ± 2.62	68	32	1
		*p* > 0.05				*p* > 0.05			*p* > 0.05
Province	Lubusz	1.66 ± 2.00	39	29	1.04 (0.61–1.97)	2.34 ± 2.59	46	22	0.72 (0.38–1.36)
	Greater Poland	1.46 ± 1.85	77	63	1	2.79 ± 2.53	104	36	1
		*p* > 0.05			*p* > 0.05	*p* > 0.05			*p* > 0.05
Socioeconomic status (in the child’s opinion)	very good	1.71 ± 1.94	58	36	1.56 (0.89–2.71)	2.36 ± 2.32	66	28	0.84 (0.46–1.55)
	average or sufficient	1.37 ± 1.85	58	56	1	2.87 ± 2.72	84	30	1
		*p* > 0.05			*p* > 0.05	*p* > 0.05			*p* > 0.05
Visitors of private dental office	yes	1.59 ± 1.97	67	53	1.02 (0.58–1.75)	2.43 ± 2.35	83	37	0.70 (0.38–1.31)
	no	1.43 ± 1.80	49	39	1	2.92 ± 2.80	67	21	1
		*p* > 0.05			*p* > 0.05	*p* > 0.05			*p* > 0.05
Daily consumption of raw vegetables and fruit	yes	1.59 ± 1.90	67	59	0.77 (0.43–1.34)	2.57 ± 2.52	91	35	1.01 (0.55–1.88)
	no	1.48 ± 1.90	49	33	1	2.74 ± 2.62	59	23	1
		*p* > 0.05			*p* > 0.05	*p* > 0.05			*p* > 0.05
Daily consumption of sweets (including cookies, cakes, candies, and donuts)	yes	1.48 ± 1.89	39	34	0.86 (0.49–1.53)	2.86 ± 2.57	53	20	1.04 (0.55–1.96)
	no	1.55 ± 1.91	77	58	1	2.52 ± 2.54	97	38	1
		*p* > 0.05			*p* > 0.05	*p* > 0.05			*p* > 0.05
Snacking sugar-containing products at the bedtime	yes	1.51 ± 1.54	80	58	1.30 (0.73–2.32)	2.75 ± 2.61	102	36	1.30 (0.69–2.44)
	no	1.54 ± 2.01	36	34	1	2.41 ± 2.45	48	22	p > 0.05
Drinking sweetened juices	several times/day	1.85 ± 2.32	14	6	1.97 (0.72–5.34)	3.25 ± 2.69	17	3	2.34 (0.66–8.32)
	up to once a day	1.49 ± 1.85	102	86	1	2.57 ± 3.54	133	55	1
		*p* > 0.05			*p* > 0.05	*p* > 0.05			*p* > 0.05
Daily consumption of sweet carbonated beverages	yes	1.74 ± 2.09	26	17	1.27 (0.64–2.53)	3.33 ± 2.76	34	9	1.60 (0.71–3.58)
	no	1.47 ± 1.84	90	75	1	2.46 ± 2.48	116	49	1
		*p* > 0.05			*p* > 0.05	*p* = 0.0477 **			*p* > 0.05
Drinking water or unsweetened tea	several times/day	1.44 ± 1.88	50	48	0.69 (0.40–1.20)	2.29 ± 2.44	65	33	0.58 (0.31–1.07)
	less frequently	1.60 ± 3.67	66	44	1	2.95 ± 2.62	85	25	1
		*p* > 0.05			*p* > 0.05	*p* > 0.05			*p* = 0.0803 ***
Milk without sugar, natural yogurts	several times/day	1.37 ± 1.88	16	14	0.89 (0.41–1.94)	3.17 ± 2.63	24	6	1.65 (0.64–4.27)
	maximum once a day	1.55 ± 3.62	100	78	1	2.55 ± 2.54	126	52	1
		*p* > 0.05			*p* > 0.05	*p* > 0.05			*p* > 0.05
Daily consumption of unsweetened chewing gums	yes	1.50 ± 1.67	22	16	2.11 (0.55–2.26)	3.11 ± 2.50	32	6	2.35 (0.93–5.96)
	no	1.53 ± 1.95	94	76	1	2.54 ± 2.56	118	52	1
		*p* > 0.05			*p* > 0.05	*p* > 0.05			*p* > 0.05
Number of meals/daily	up to 3	1.41 ± 1.77	15	16	1.42 (0.66–3.05)	2.39 ± 2.40	22	9	0.94 (0.40–2.17)
	>3	1.54 ± 1.92	101	76	1	2.68 ± 2.58	128	49	1
		*p* > 0.05			*p* > 0.05	*p* > 0.05			*p* > 0.05
Snacking between 5 main meals	yes	1.45 ± 1.78	68	55	1.95 (0.55–1.66)	2.89 ± 2.65	85	38	0.69 (0.37–1.29)
	no	1.63 ± 2.06	48	37	1	2.46 ± 2.48	65	20	1
		*p* > 0.05			*p* > 0.05	*p* > 0.05			*p* > 0.05
Frequency of toothbrushing	less frequently	1.55 ± 1.96	48	40	0.92 (0.53–1.60)	3.39 ± 2.81	70	18	1.94 (1.02–3.70)
	at least twice/day	1.51 ± 1.85	68	52	1	2.09 ± 2.20	80	40	1
		*p* > 0.05			*p* > 0.05	p = 0.0003 **			*p* = 0.0424 ****
Frequency of toothbrushing	less frequently	1.63 ± 1.95	13	11	0.93 (0.40–2.18)	4.54 ± 2.84	23	1	10.32 (1.36–78.32)
	at least once/day	1.51 ± 1.89	103	81	1	2.39 ± 2.41	127	57	1
		*p* > 0.05			*p* > 0.05	*p* = 0.0001 **			*p* = 0.0240 ****
The use of dental floss	yes	1.67 ± 2.20	46	35	1.07 (0.61–1.88)	2.62 ± 2.70	59	22	1.06 (0.57–1.98)
	no	1.43 ± 1.68	70	57	1	2.65 ± 2.47	91	36	1
		*p* > 0.05			*p* > 0.05	*p* > 0.05			*p* > 0.05
The use of fluoridated toothpaste	yes or I am not sure	1.49 ± 1.85	88	109	1.41 (0.40–4.98)	2.58 ± 2.48	142	55	0.99 (0.25–3.78)
	no	2.09 ± 2.70	4	7	1	3.73 ± 3.61	8	3	1
		*p* > 0.05			*p* > 0.05	*p* > 0.05			*p* > 0.05
Participation in school-based program of supervised toothbrushing with fluoride gel	yes	1.65 ± 2.04	52	75	0.71 (0.41–1.25)	2.75 ± 2.62	95	32	1.40 (0.76–2.60)
	no or I do not remember	1.32 ± 1.64	40	41	1	2.47 ± 2.45	55	26	1
		*p* > 0.05			*p* > 0.05	*p* > 0.05			*p* > 0.05

DMFt: the number of decayed, missing, filled teeth; ICDASepiDMTt: the DMTt index including incipient caries lesions; * a close to significant difference; ** a statistically significant difference; *** a close to significant association, and **** a statistically significant association.

**Table 6:** The association between oral hygiene and dietary habits in the examined adolescents.

Factor	Categories of Response	Frequency of Toothbrushing		The Odds Ratio of Less Frequent Toothbrushing	
		At Least Twice/Day	Maximum Once/Day		*p* Value
		n (%)	n (%)	OR (95% CI)	
Daily consumption of raw vegetables and fruit	yes	81	45	1.99 (1.13-3.50)	*p* = 0.0177 *
	no	39	43	1	
Daily consumption of sweets (including cookies, cakes, candies, and donuts)	yes	37	36	1.55 (0.87–2.76)	*p* < 0.05
	no	83	52	1	
Snacking on sugar-containing products at bedtime	yes	65	73	0.24 (0.13–0.47)	*p* < 0.0001 *
	no	55	15	1	
Drinking sweetened juices	several times/day	9	11	0.57 (0.23–1.44)	*p* < 0.05
	up to once a day	111	77	1	
Daily consumption of sweet carbonated beverages	yes	20	23	0.57 (0.29–1.11)	*p* = 0.0979 **
	no	100	20	1	
Drinking water or unsweetened tea	several times/day	64	34	1.82 (1.04–3.18)	*p* = 0.0367 *
	less frequently	56	54	1	
Milk without sugar, natural yogurts	several times/day	18	12	1.12 (0.51–2.46)	*p* < 0.05
	maximum once a day	102	76	1	
Daily consumption of unsweetened chewing gums	yes	22	16	1.01 (0.50–2.06)	*p* < 0.05
	no	98	72	1	
Number of meals/daily	up to 3	15	16	0.64 (0.30–1.38)	*p* < 0.05
	>3	105	72	1	
Snacking between the five main meals	yes	67	56	0.72 (0.41–1.27)	*p* < 0.05
	no	53	32	1	
The use of dental floss	yes	56	25	2.21 (1.23–3.96)	*p* = 0.0082 **
	no	64	63	1	

* statistically significant association, ** close to significant association.

## Data Availability

The datasets generated for this research are available from the corresponding author on reasonable request.
